# Using pollen DNA metabarcoding to profile nectar sources of urban beekeeping in Kōtō-ku, Tokyo

**DOI:** 10.1186/s13104-020-05361-2

**Published:** 2020-11-10

**Authors:** Keisuke Tanaka, Akinobu Nozaki, Hazuki Nakadai, Yuh Shiwa, Mariko Shimizu-Kadota

**Affiliations:** 1 NODAI Genome Research Center, Tokyo University of Agriculture, Setagaya, Tokyo, 156-8502 Japan; 2 Graduate School of Environmental Sciences, Musashino University, Kōtō-ku, Tokyo, 135-8181 Japan; 3 Department of Environmental Sciences, Musashino University, Kōtō-ku, Tokyo, 135-8181 Japan; 4 Department of Molecular Microbiology, Faculty of Life Sciences, Tokyo University of Agriculture, Setagaya, Tokyo, 156-8502 Japan; 5 Department of Environmental Systems Sciences, Faculty of Engineering, Musashino University, Kōtō-ku, Tokyo, 135-8181 Japan; 6 Musashino University Creating Happiness Incubation, Musashino University, Kōtō-ku, Tokyo, 135-8181 Japan

**Keywords:** Honeybee, Urban beekeeping, Nectar source, Pollen, DNA metabarcoding, Metagenome, Ribulose 1,5-bisphosphate carboxylase/oxygenase, Chloroplast, Next generation sequencing

## Abstract

**Objective:**

*Apis mellifera* is a species of honeybee that has been introduced around the world as an industrial beekeeping species. Recently, urban beekeeping has attracted attention as a means of ecosystem protection and urban greening. This study aimed to investigate nectar sources of urban beekeeping in Kōtō-ku, Tokyo using pollen DNA metabarcoding.

**Results:**

We extracted DNA from pollen collected by the honeybees of a local urban beekeeping operation. DNA metabarcoding analysis was carried out by sequencing a part of the *rbc*L region of the chloroplast genome. A total of 31 samples collected between mid-March, 2018 and mid-October, 2018 yielded 54 operational taxonomic units (OTUs) comprising 14 families, 32 genera, and 8 species. Whereas 5 OTUs were profiled throughout all seasons, 38 OTUs were season-specific (spring, summer, or autumn). Therefore, we were able to infer seasonal nectar sources for the beekeeping operation at the family or genus level, as well as at the species level to a lesser extent. Our pollen-sampling strategy was effective for profiling season-specific nectar sources, with the exception of a few anomalies that can be accounted for by out-of-season flowering associated with artificial gardening and/or pollen accumulation over multiple seasons.

## Introduction

*Apis mellifera* is a honeybee species that has been introduced around the world as an industrial beekeeping species. Although *Apis cerena* has long been used in Japanese beekeeping as a part of the traditional culture, *A*. *mellifera* was introduced via the United States in 1877 [1]. Honeybees generally collect nectar and pollen from flowers that they visit to provide the nutrients necessary for colony maintenance and development [2]. Nectar is processed to form honey, the main energy source for the colony. Pollen represents the colony’s only supply of protein and is essential for brood rearing and the development of hypopharyngeal glands in young worker bees [3]. Urban beekeeping has recently attracted worldwide attention as a useful method of bee conservation in urban areas and as a method to promote urban greening, which is an important countermeasure to heat islands [4–6]. A Japanese urban beekeeping project known as the Ginza Honey Bee Project was initiated in Tokyo in 2006 and is currently being implemented in many cities and communities [7]. In addition to honey harvesting, this project includes four objectives as follows: environmental education, such as providing honey harvesting experiences and workshops; development of local brands using the harvested honey; promotion of urban greening around the region; investigation of surrounding nectar and pollen sources [8].

Since high-throughput sequence technology using next-generation sequencing (NGS) appeared recently, a DNA metabarcoding technique based on metagenomic analysishas enabled investigation into various aspects of biome compositions by comprehensively identifying barcode regions common to organisms occupying a particular habitat type (e.g. soil, water, or air). Conventional DNA barcoding based on Sanger sequencing technology has been used to analyse pollen collected by honeybees [9–11]. While this technique can be useful in elucidating information on a fine scale, it is not practical for large-scale application [12]. To address this limitation, DNA metabarcoding has recently been applied to pollen collected by honeybees [13–16]. This updated approach to pollen analysis is expected to yield improvements in efficiency, cost and labour.

**Fig. 1:**
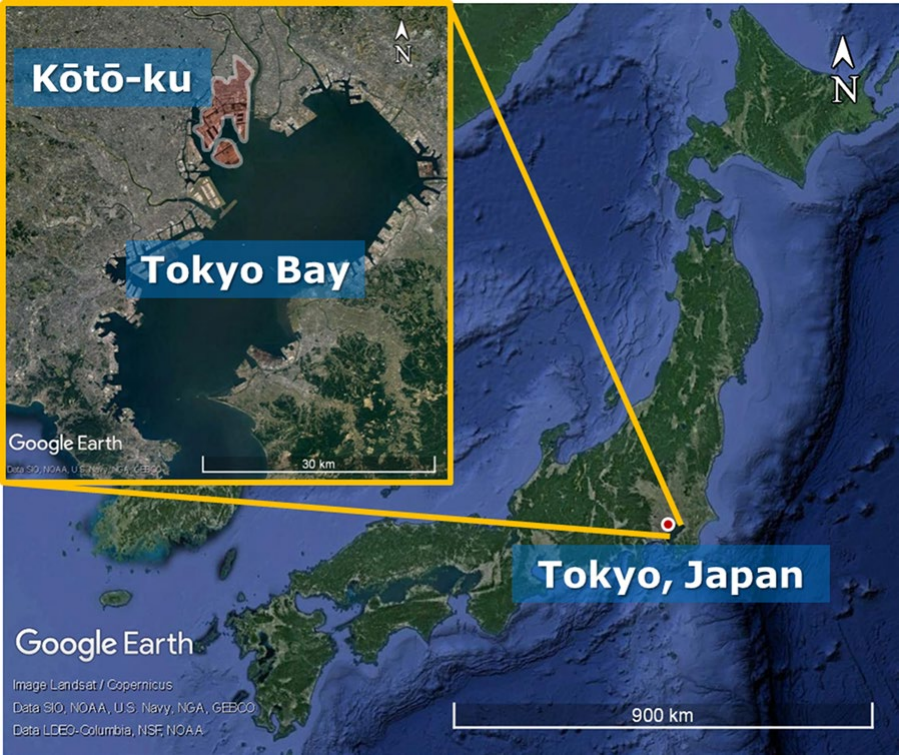
Topography of Kōtō-ku, located on the waterfront of Tokyo Bay, Japan

Located on the waterfront of Tokyo Bay, Kōtō-ku is a special ward within Tokyo Metropolis, Japan, and has been targeted for development as a green city (Fig. 1). Urban beekeeping has been practised at Musashino University of Kōtō-ku since 2014 as a practical education program that is part of the ‘Living Laboratory for Sustainability’ environmental project. This project explores the potential role of urban beekeeping as an integral aspect of a sustainable landscape design model based on symbiosis among honeybees, plants, and humans. However, knowledge of surrounding nectar-source plants is primary to successful beekeeping. By using DNA metabarcoding to analyse pollen collected by the honeybees, this study investigated the kinds of plants that serve as nectar sources for the Musashino University bee colonies, because honeybees collect both nectar and pollen as described.

## Methods

### Materials collection

Beekeeping (*A. mellifera*) is carried out on the roof of the second building at Musashino University Ariake Campus in the Ariake district of Kōtō-ku, on the Tokyo Bay coast (Fig. 1). A microspatula-tip full of bee pollen was obtained from an uncovered and relatively new honeycomb. Pollen was bright orange or yellow in colour, with low viscosity and low permeability. The honeybee in temperate regions including Japan has a foraging season, which is spring to autumn [9]. Samples were collected three times per month (representing early, middle, and late periods) on rain-free days during daylight hours from mid-March, 2018 to mid-October, 2018, with the exception of the mid-July sample period, because of honeybee behavioural suppression with continuous extreme heat days (Additional file 1, Fig. S1).

### DNA metabarcoding analysis

After adding Lysis Solution F (Nippon Gene, Tokyo, Japan), 0.5-mm zirconia beads, and 5.0-mm stainless steel beads to the bee pollen sample, the liquid was shaken at 1500 rpm for 2 min using a Shake Master Neo (Biomedical Science, Tokyo, Japan) and was then incubated at 65 °C for 10 min. After centrifugation at 12,000×*g* for 10 min, supernatant was collected. Genomic DNA was extracted using an MPure Bacterial DNA Extraction Kit (MP Bio Japan, Tokyo, Japan). The extracted DNA solution was mixed with a final concentration of 10% polyvinylpolypyrrolidone and purified by collecting the supernatant after centrifugation. The DNA concentration was measured with a Synergy H1 (BioTek, Winooski, VT, USA) and a QuantiFluor dsDNA System (Promega, Madison, WI, USA).

An amplicon library targeting a part of the *rbc*L region of the chloroplast genome was constructed by two-step PCR. The 1st PCR primer set was customised within the barcode region as a mini-barcoding system for amplicon sequencing by short-read NGS [17]. This PCR was carried out in a total volume of 10 μl, containing 0.5 ng template DNA, 0.5 μM forward primer (5ʹ-ACACTCTTTCCCTACACGACGCTCTTCCGATCTCTTACCAGYCTTGATCGTTACAAAGG-3ʹ [underline indicates the Illumina adapter sequence]), 0.5 μM reverse primer (5ʹ-GTGACTGGAGTTCAGACGTGTGCTCTTCCGATCTGTAAAATCAAGTCCACCRCG-3ʹ [underline indicates the Illumina adapter sequence]), 0.2 mM dNTP mixture, 1 × company-supplied buffer, and 0.05 U ExTaq HS DNA polymerase (TaKaRa Bio, Shiga, Japan). The reaction cycles were as follows: initial denaturation at 94 °C for 2 min; 30–35 reaction cycles at 94 °C for 30 s, 50 °C for 30 s, and 72 °C for 30 s, and final extension at 72 °C for 5 min. After clean-up of the 1st PCR product, the 2nd PCR was performed under the same conditions, with the following modifications: a forward primer (5ʹ-AATGATACGGCGACCACCGAGATCTACAC[index 2]ACACTCTTTCCCTACACGACGC-3ʹ) and reverse primer (5ʹ-CAAGCAGAAGACGGCATACGAGAT[index 1]GTGACTGGAGTTCAGACGTGTG-3ʹ) were added based on the Illumina Adapter Sequences Document (https://support.illumina.com/content/dam/illumina-support/documents/documentation/chemistry_documentation/experiment-design/illumina-adapter-sequences-1000000002694-14.pdf), and adjustments were made to some PCR programs (12 cycles and annealing temperature at 60 °C). After clean-up of the 2nd PCR product, the quality of the constructed library was checked using a Fragment Analyzer (Advanced Analytical Technologies, Ankeny, IA, USA) and a dsDNA 915 Reagent Kit (Advanced Analytical Technologies). Multiple libraries were pooled and sequenced by running 2 × 300-bp paired-end reads using a MiSeq platform (Illumina, San Diego, CA, USA).

A series of bioinformatic analyses is shown in Additional file 2, Fig. S2. Briefly, raw read data were cleaned by removing primer sequences using the Fastx-Toolkit version 0.0.14 (https://hannonlab.cshl.edu/fastx_toolkit/). In addition, reads with a quality score of less than 20 or length less than 40 nt were excluded using Sickle version 1.33 (https://github.com/ucdavis-bioinformatics/sickle). The clean paired reads were merged using FLASh version 1.2.11 [18]. Parameters were merged as follows: (i) minimum overlap length = 10, (ii) average read length = 230, and (iii) average fragment length = 320. Sequence dereplication, sorting by decreasing abundance, operational taxonomic unit (OTU) clustering, chimera filtering, and mapping reads back to OTUs were performed using USEARCH version 10.0.240 (https://www.drive5.com/usearch/). After reads were mapped to representative OTUs, they were normalised by counts per million (CPMs). The most abundant sequence from each OTU was selected as the representative sequence and was annotated for target species with 97% similarity against the NCBI non-redundant nucleotide ‘nt’ database using the blastn program (BLAST+ version 2.7.1) [19]. For each OTU that was annotated to different species with the same similarity score, the genus or family common to those was assigned.

## Results

A total of 31 samples were obtained from each sampling period. Thirteen samples (for example early May) were taken from a single honeycomb on the same day to assess any potential variation in pollen accumulated among different honeycombs. Sequence data for 6994–95,832 paired-end reads were generated as output for each sample (Additional file 3, Table S1). Approximately 13–94% reads per sample were available for OTU profiling. Non-target OTUs, which included sequences that were classified as honeybee, human, *Zygosaccharomyces* sp., shuttle vector, *Ralstonia pickettii*, and *Trebouxia showmanii*, were excluded from analysis. Those classified as Pinaceae, Cupressaceae, Podocarpaceae, Arecaceae, Poaceae, and Woodsiaceae were also excluded, as these represent wind-pollinated flowers.

**Fig. 2:**
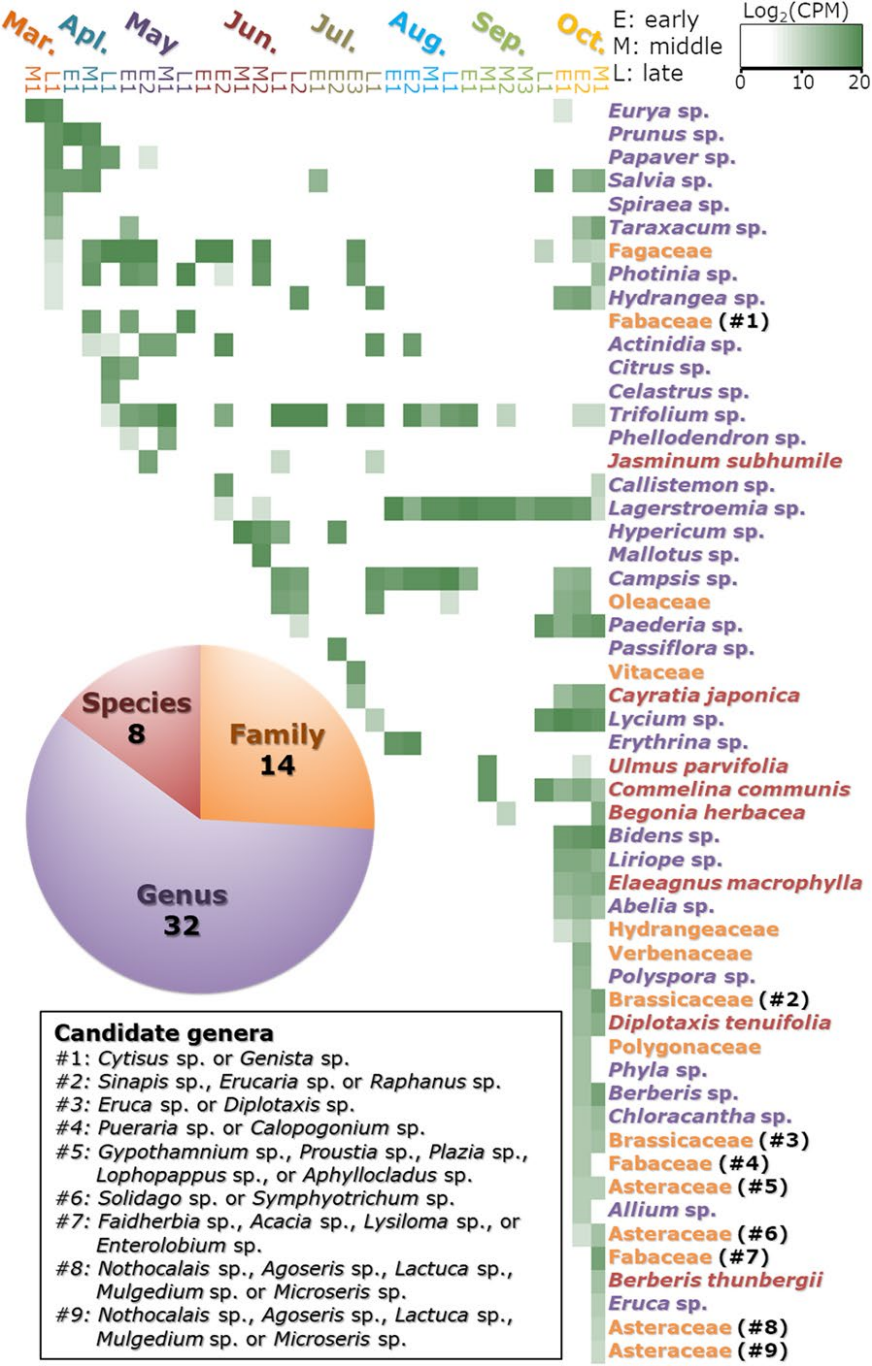
Profiles of annotated operational taxonomic units (OTUs) from each sample. The read count for each OTU is indicated by the log_2_CPM (counts per million). Taxonomic level is indicated by colour: family (orange), genus (purple), and species (red). These categories are shown as pie charts in the lower left portion of the figure. To facilitate visualisation of variations in seasonal patterns, the annotated OTUs are listed in the order of appearance from March to October. Nine OTUs with the symbol “#” show that they are annotated as different species with the same similarity score; candidate genera are described within this figure

A total of 54 OTUs were obtained by DNA metabarcoding analysis (Fig. 2). They were annotated as comprising 14 families, 32 genera, and 8 species. The average number of OTUs per sample was 5.9, with a range of 1–31. Moreover, multiple samples that were obtained from different honeycombs on the same day shared some OTUs in common with each other, although the matches were imperfect. The family Fagaceae and the genera *Salvia*, *Photinia*, *Hydrangea*, and *Trifolium* were detected throughout all three seasons. The family Fabaceae and the genera *Prunus*, *Papaver*, *Spiraea*, *Citrus*, *Celastrus*, and *Phellodendron* were detected only in spring (March, April, and May). The family Vitaceae and the genera *Hypericum*, *Mallotus*, *Passiflora*, and *Erythrina* were detected only in summer (June, July, and August). The families Brassicaceae, Fabaceae, Hydrangeaceae, Verbenaceae, Asteraceae, and Polygonaceae, the genera *Bidens*, *Liriope*, *Abelia*, *Polyspora*, *Phyla*, *Berberis*, *Chloracantha*, *Allium*, and *Eruca*, and the species *Ulmus parvifolia*, *Commelina communis*, *Begonia herbacea*, *Elaeagnus macrophylla*, *Diplotaxis tenuifolia*, and *Berberis thunbergii* were detected only in autumn (September and October).

## Discussion

Although our approach enabled profiling of many families and genera, few identifications were made at the species level. In particular, half of the OTUs annotated at the family level were related to Asteraceae and Fabaceae, which constitute two of the three largest plant families (the third being Orchidaceae) [20]. The restrictive approach to barcoding used here, which was specific to the *rbc*L region of the chloroplast genome, might lead to difficulty in recognising common sequences between species. However, an even more complex approach, which uses portions from both the *rbc*L and *mat*K regions of the chloroplast genome along with the ITS2 region of the nuclear genome, has not been completely refined [16]. Therefore, the technique of applying DNA metabarcoding to plants using pollen requires further improvement in terms of the identification and selection of more robust barcoding regions, as well as the accrual of many more reference genomes.

In this study, five OTUs were detected in all three seasons. These included garden plants such as sage (*Salvia* sp.) and white clover (*Trifolium* sp.), which flower throughout the entire year. In contrast, 38 OTUs were detected as ‘seasonal OTUs’. These include park and street plants such as cherry (*Prunus* sp.) and echidna (*Cytisus* sp.) in spring, Mallotus bark (*Mallotus* sp.) and passion flower (*Passiflora* sp.) in summer, and lacebark elm (*Ulmus parvifolia*) and broad-leaved oleaster (*Elaeagnus macrophylla*) in autumn. However, based on profiled OTUs, some plants were suggested to be present in seasons other than their typical flowering time. In particular, samples collected during the autumn tended to yield many such annotations. This could possibly be the result of two factors, out-of-season flowering induced by artificial gardening techniques and/or pollen accumulation over multiple seasons. As noted, we found that different honeycombs sampled on the same day tended to share some OTUs. However, our sampling method might require improvement, because it allowed arbitrariness in sample selection on the part of the experimenter. One potential improvement could be the use of a pollen trap [21]. We can therefore conclude that the sampling method is a very important consideration in pollen DNA metabarcoding analysis.

**Fig. 3:**
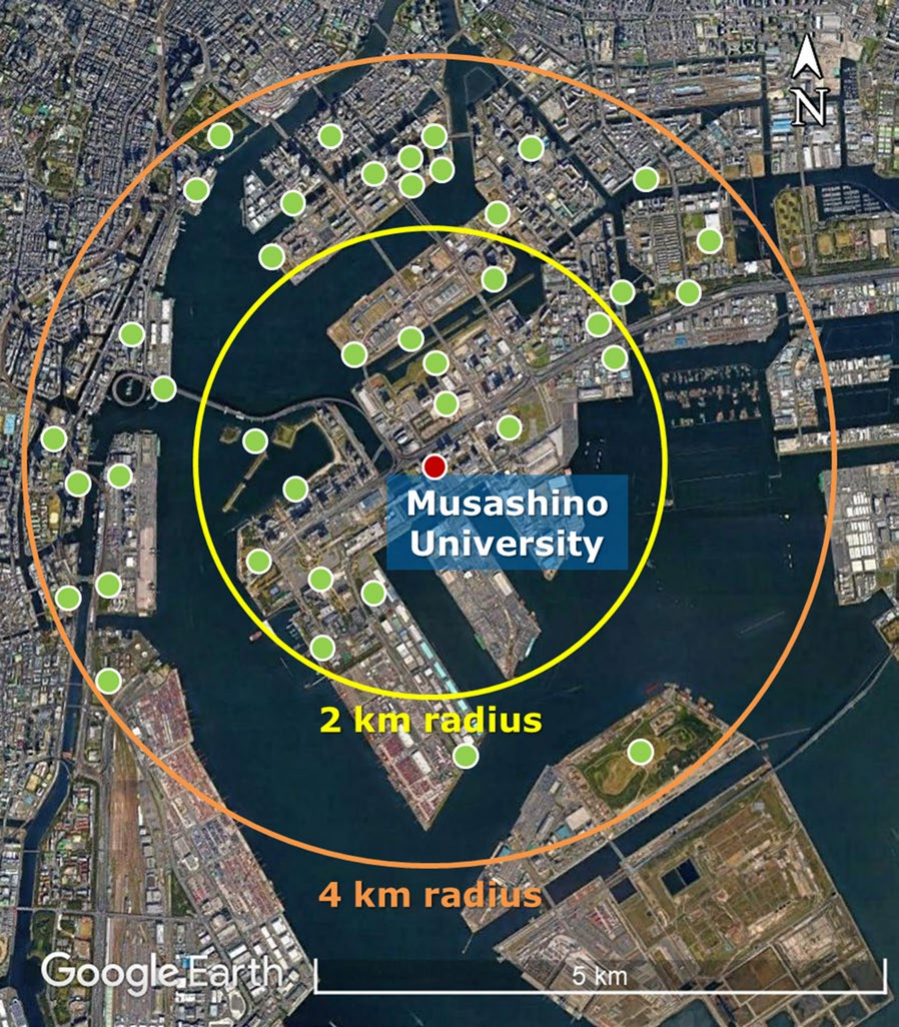
Locations of major planted parks and grounds in Kōtō-ku. The red plot shows the beekeeping site at Musashino University; the green plot shows parks or grounds

The range of honeybee travel has long been believed to be within 2 km [22]. However, recent reports based on new research techniques indicate that bees can forage approximately 10 km further than previously thought [23, 24]. A large variety of plants occupies the 14 parks or grounds that occur within a radius of 2 km from our beekeeping site (Fig. 3). In addition, 26 parks or grounds are located across the sea within a distance of 4 km. Although it is plausible that honeybees could forage from these locations, the presence of only a few OTUs indicated that this was the case. We posit that the honeybee foraging range could be limited by beekeeping management practises and inclement weather (Additional file 3, Table S2). However, this study did not demonstrate these associations.

In summary, most plants used as nectar sources by the urban honeybees studied herein were identified at the family and genus levels. Most plants could not be identified at the species level. We conclude that further development of this approach will enable the creation of a full-year pollen calendar.

### Limitations

The data used in this study were obtained through metabarcoding of plant DNA that was derived from pollen collected by honeybees. However, there is no supporting data regarding the locations of the identified plant species.

## Supplementary information

**Additional file 1: Figure S1.** Illustration of the bee hive from the apiaryused in this study. Pollen wasobtained from a relatively new honeycomb and was bright orange or yellowin colour, with low viscosity and low permeability.

**Additional file 2: Figure S2.**Flowchart of the series of bioinformaticanalyses.

**Additional file 3: Table S1.** Summary of collection date, sequence summary, and OTU profiling in each sample. **Table S2.** Meteorological observation data in Tokyo on March to October, 2018.

## Abbreviations

DNA: Deoxyribonucleic acid
NGS: Next-generation sequencer
*rbc*L: Ribulose-1,5-bisphosphate carboxylase/oxygenase
PCR: Polymerase chain reaction
OTU: Operational taxonomic unit
CPM: Counts per million
*mat*K: Maturase K
ITS2: Internal transcribed spacer 2

## Availability of data

The dataset supporting the conclusions of this article is available in the DDBJ Sequence Read Archive (accession number DRA010035) after the publication of this report.
