# Supplementary figures and images for: Using pollen DNA metabarcoding to profile nectar sources of urban beekeeping in Kōtō-ku, Tokyo
Source: BMC Res Notes. 2020 Nov 10;13:515. doi: 10.1186/s13104-020-05361-2 (PMC7653823; doi:10.1186/s13104-020-05361-2)

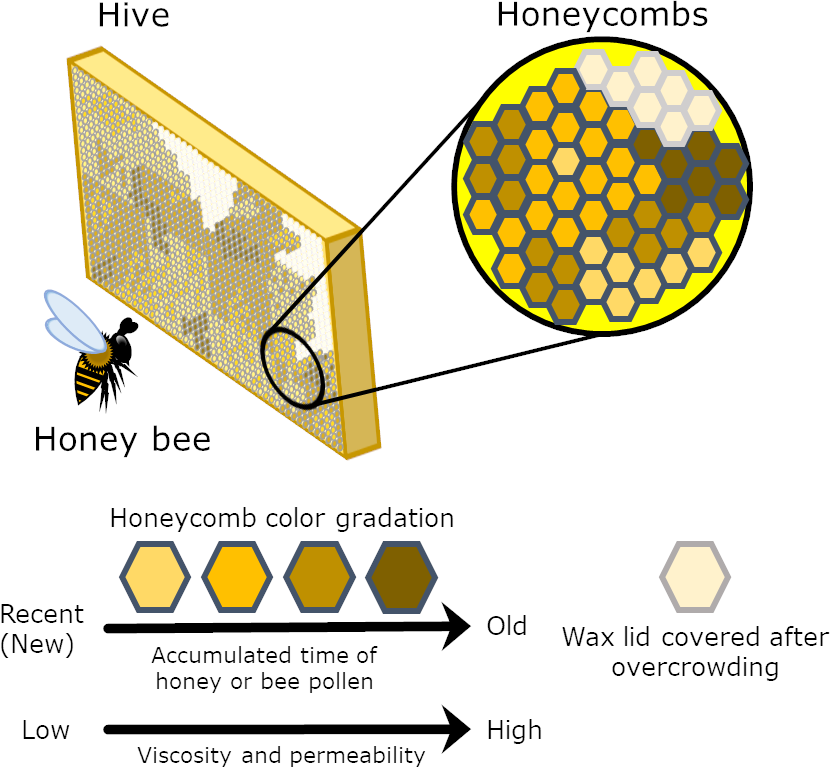

Supplement: Supplementary file 1 — Additional file 1: Figure S1. Illustration of the bee hive from the apiaryused in this study. Pollen wasobtained from a relatively new honeycomb and was bright orange or yellowin colour, with low viscosity and low permeability. [file 13104_2020_5361_MOESM1_ESM.png]

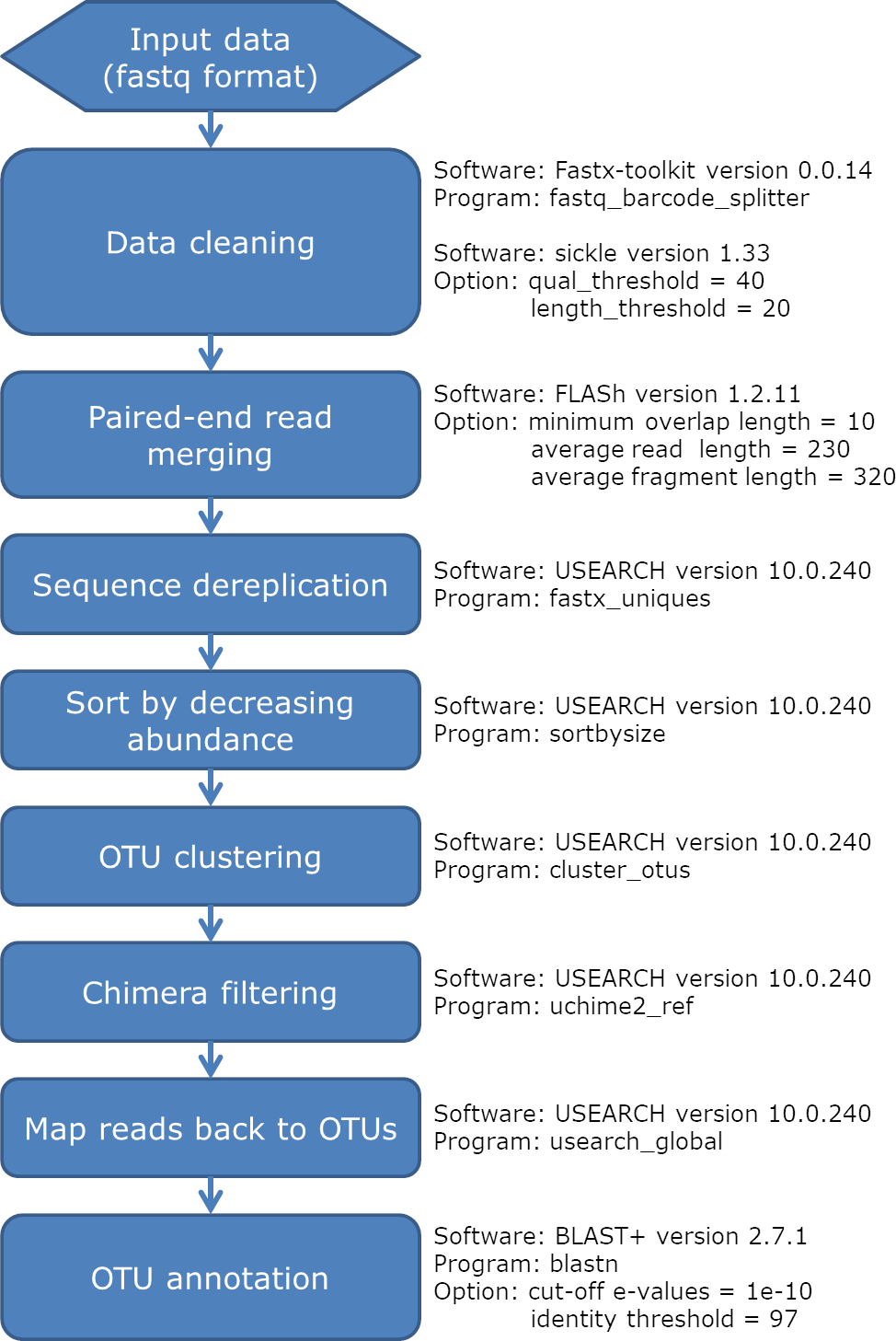

Supplement: Supplementary file 2 — Additional file 2: Figure S2.Flowchart of the series of bioinformaticanalyses. [file 13104_2020_5361_MOESM2_ESM.png]
